# Left Ventricular Non-Compaction Cardiomyopathy: The Tragedies & Trabeculations of the Architectural Cardiac Sponge

**DOI:** 10.3390/jcm15052023

**Published:** 2026-03-06

**Authors:** Noyan Ramazani, Brooke Ivey, Shudipan Chakraborty, Daniel Bishev, Michael DiCaro, Paul Duru, Ryan Shao, Aditi Singh

**Affiliations:** 1 Department of Internal Medicine, Kirk Kerkorian School of Medicine at University of Nevada, Las Vegas, NV 89102, USA; aditi.singh@unlv.edu; 2 Kirk Kerkorian School of Medicine at University of Nevada, Las Vegas, NV 89106, USA; iveyb1@unlv.nevada.edu; 3 Department of Cardiovascular Medicine, Kirk Kerkorian School of Medicine at University of Nevada, Las Vegas, NV 89106, USA; shudipan.chakraborty@unlv.edu (S.C.); daniel.bishev@unlv.edu (D.B.); michael.dicaro@unlv.edu (M.D.); paul.duru@unlv.edu (P.D.); ryan.shao@unlv.edu (R.S.)

**Keywords:** left ventricular noncompaction, cardiomyopathy, myocardial trabeculations, cardiac sponge, pediatric myocardium, congenital heart disease, heart failure, arrhythmia, sudden cardiac arrest, sudden cardiac death

## Abstract

Left-ventricular non-compaction (LVNC) is a recently classified cardiomyopathy that involves abnormal trabeculations inside the left ventricle, most commonly located in the ventricular apex. There are 9 distinct types of non-compaction cardiomyopathy that can impact both the left and right ventricles with subtypes involving mostly pediatric patients with concurrent congenital heart disease (CHD), to individuals in late adult-staged ages. LVNC affects the population with an estimated range of incidence from 0.014% to 1.3% and the disease can be diagnosed with the utilization of imaging studies such as transthoracic echocardiography (TTE). LVNC can also impact and lead patients to develop heart failure with estimated prevalence that can reach to 3–4% during their lifetime. LVNC often leads to complications such as heart failure, arrhythmias, and thromboembolic events and without adequate medical management and pharmacological therapies this can progress and lead to worsening cardiac function, sudden cardiac arrest, and even death. There are no strict guidelines organized for screening and monitoring for LVNC in patients except with the inclusion of having a high suspicion in patients without other cardiac abnormalities. Thus, more advanced clinical research and the establishment of diagnostic protocols needs to be standardized in order to further investigate the causes, prognostic factors and therapeutic modalities of patients with LVNC. The field of LVNC cardiomyopathy is expanding but better understanding of the pathophysiology and genetic influence of this cardiac disease is vital for the precision treatment and personalized care of LVNC.

## 1. Introduction

**Figure 1:**
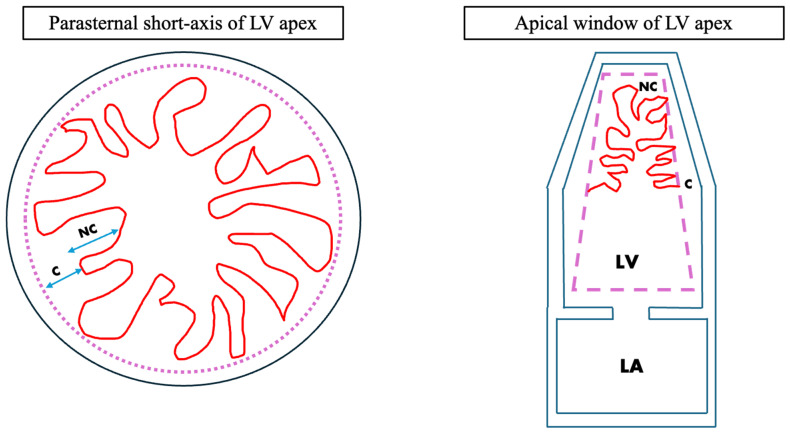
Illustrative depiction of the LV with trabeculations in the setting of NC vs. C myocardial layers. **NC** = non-compaction myocardium, **C** = compacted myocardium, **LA** = left-atrium, **LV** = left-ventricle.

Left ventricular noncompaction (LVNC) cardiomyopathy is caused by the development of prominent myocardial trabeculations resembling a sponge (“spongy myocardium”) and deep recesses commonly seen inside the left ventricle (LV) which are composed of a two-layered myocardium consisting of a noncompacted (NC) and a compacted (C) layer [1,2] (Figure 1). In 2006, the American Heart Association (AHA) Scientific Statement categorized LVNC as a formal cardiomyopathy while the European Working Group of Myocardial Disease considers LVNC as merely a trait rather than a distinct cardiomyopathy [3,4]. LVNC cardiomyopathy can also affect the right ventricle (RV), or both ventricles simultaneously which can lead to progressive ventricular systolic dysfunction leading to fatal arrhythmias and possibly to the creation of systemic thromboembolic events as a consequence [5,6]. Some etiologies of LVNC can be associated with malformations or deformations arising from congenital heart diseases (CHD) involving improper embryological cellular signaling, metabolic, mitochondrial, and even neuromuscular and other genetic diseases [6]. The excessive trabeculations seen in LVNC are believed to occur as a consequence of molecular checkpoint arrest in the compaction phase of fetal development, this step is crucial for the compact myocardium to form [7].

LVNC is not classified as a distinct, independent cardiomyopathy by the European Society of Cardiology (ESC) but rather as an unclassified cardiomyopathy or a “phenotypic trait,” that can coexist with or mimic other true bona fide cardiomyopathy types. Several gene mutations related to LVNC have been identified including sarcomeric, cytoskeletal, Z-line, ion channel, mitochondrial, and signaling proteins based on evidence provided by the ESC and the AHA [8]. The ESC has differentiated clear terminology when comparing hypertrabeculations versus LVNC emphasizing it as an architectural structural trait which can be found in healthy individuals such as in athletes or found as a secondary feature in other heart diseases.

The controversy surrounding the universal acceptance of categorizing LVNC as a distinct cardiomyopathy rather than a variant of another disease state is still up for debate between various international governing institutions and academic organizations. The contents of this framework make arguments for both sides in whether LVNC is categorically a type of cardiomyopathy or an adaptive, remodeling phenotype seen in various demographic populations (i.e., pregnancy and athletes) and only serving as a mere physiologic structure. This manuscript will highlight the types of LVNC cardiomyopathy, discuss statistics in how it pertains to the epidemiology of the disease, and provide a detailed assessment of the risk factors, genetic components and pathophysiology interplay leading to the complications that arise. Lastly, this manuscript will discuss the diagnostic imaging studies needed to portray LVNC cardiomyopathy and the treatment modalities utilized to decrease morbidity and mortality.

## 2. Materials & Methods

We conducted a thorough search in medical databases such as PubMed, Embase, and Google Scholar for biomedical research articles, pertinent trials, and clinical registries pertaining to left ventricular non-compaction (LVNC) cardiomyopathy and other related keyword variants.

## 3. Types of LVNC Cardiomyopathy

LVNC does not have a unified and majority accepted classification system but one that is rooted in making the types of LVNC cardiomyopathy more organized and consolidated in hopes that the information available on this specific cardiac disease is easily accessible. Thus, the types of LVNC cardiomyopathy that will be provided in this text are merely an attempt to better grasp and understand LVNC cardiomyopathy and categorize each type into a specific category. Various research papers have indicated that LVNC presents with nine various phenotypic subsets or heterogeneities that are summarized in Table 1 and those are:

### 3.1. Type I LVNC

The first subtype of LVNC involves isolated LV hyper-trabeculation without any other physiological features of heart disease which traditionally involves the change in LV size, thickness, and/or systolic and diastolic dysfunction [7]. The first subtype also has the absence of other structural heart diseases and does not possess any malfunctions in the electrical conduction mechanism of the heart which can commonly cause arrhythmias, and thus the first subtype is also termed the “isolated form of LVNC” [7]. This subgroup is often referred to as benign LVNC and counts as approximately 25–35% of all childhood patients and overall, these patients generally do well. However, it was discovered that this subtype of LVNC in neonates can also present with echocardiography findings consistent with LVNC with systolic dysfunction but with normal LV dimensions which almost presents like a version of Type I LVNC but shares some features of this heterogeneity of LVNC with some minor nuances [9]. The prognosis impact and management of type I LVNC is seen as favorable, as isolated LVNC in patients presented with little to no cardiovascular deaths which was observed in long-term follow-up studies [10]. Compared to the dilated type of LVNC, isolated LVNC is shown to have better quality of life and less risk of developing severe morbidity and mortality.

### 3.2. Types II, III, & IV LVNC

The second subtype of LVNC is frequently termed arrhythmogenic LVNC cardiomyopathy which places these patients at a high risk of syncope, sudden cardiac arrest (SCA), and sudden cardiac death (SCD) from deadly arrhythmias caused by ventricular tachycardia, ventricular fibrillation, atrioventricular block, and supraventricular tachycardia, like atrial fibrillation [7]. The third subtype of LVNC includes non-compaction and dilated cardiomyopathy (DCM) and when both are combined, they create worse outcomes as a result of the dilated hyper-trabeculations of the LV in addition to a depressed systolic function [7]. The fourth subtype of LVNC involves the inclusion of hypertrophic cardiomyopathy (HCM) and causes hypercontractile systolic function and diastolic dysfunction with a small, hyper-trabeculated LV cavity due to the thickening of the cardiomyocytes [7].

Prognosis and complication development in types II, III, and IV LVNC are all related to the development of common arrhythmias such as atrial fibrillation which impacts 25–30% of patients with this particular LVNC type [11]. Arrhythmogenic LVNC often worsens systolic function and creates an environment that is conducive to a higher prevalence of atrioventricular valve regurgitation as a consequence of this type of LVNC [11]. LVNC types II, III, and IV also foster an environment that encourages high risk thrombi to form thereby increasing the dangers of cerebrovascular accidents to occur, often requiring anticoagulation to prevent neurological complications [11]. Other scenarios that can be seen in this LVNC type include the rise of severe and deadly ventricular tachyarrhythmias that can lead to sudden cardiac arrest and even sudden cardiac death. An implanted cardiac defibrillator (ICD) is one tool used to prevent devastating and deadly arrhythmias in this LVNC type and is often recommended in those with an ejection fraction (EF) lower than 35% [11].

### 3.3. Type V LVNC

The fifth subtype of LVNC consists with HCM plus DCM, isolated HCM, or isolated DCM with LVNC associated with a restrictive hemodynamic physiology often termed “undulating phenotype” or mixed form of LVNC which goes from hypertrophy to dilated back to hypertrophic disease until cardiomyocytes have been physiologically exhausted [7]. The end-stage of the fifth subtype of LVNC causes DCM over a relatively short period of time in the pediatric population and as such these patients tend to present with heart failure or arrhythmias, especially if they already have a prior or concurrent metabolic or mitochondrial dysfunction [7]. The dilated subtype of LVNC has been shown and associated to create the most complicated prognostic factors and is often interlinked with other cardiovascular disorders like hypertension, diabetes mellitus, dyslipidemia, and coronary artery disease [10].

### 3.4. Types VI & VII LVNC

The sixth subtype of LVNC is often termed the restrictive cardiomyopathy version of LVNC that involves atrial dilation with concomitant diastolic dysfunction associated with normal LV dimension, thickness, systolic function without any mitral valve regurgitation making this subtype a rare but high-risk and high-morbidity subtype of LVNC [7]. The seventh form of LVNC is called the biventricular cardiomyopathy LVNC where both the LV and RV are hyper-trabeculated and both systolic and diastolic dysfunction are commonly seen. Similar to subtype six of LVNC, subtype seven is also uncommon and rarely found in the pediatric population [7].

### 3.5. Types VIII & IX LVNC

The eight subtype of LVNC is actually a misnomer because it involves RV non-compaction (RVNC) which is rare and causes RV hyper-trabeculation while the LV appears normal [7]. The ninth and last subtype of LVNC is seen to commonly affect congenital heart disease (CHD) forms of LVNC which is frequently seen and associated with right-heart obstructive disease [7].

## 4. Epidemiology

The epidemiology of left ventricular noncompaction cardiomyopathy (LVNC) remains a developing area of study. Historically considered a rare congenital anomaly, LVNC is now diagnosed more frequently, largely due to advances in cardiac imaging and broader screening of patients at genetic risk or with suggestive cardiac symptoms [12,13]. While this rise in detection has helped increase awareness of LVNC, it has also made it more difficult to define and classify the condition consistently. Reported prevalence varies widely, largely depending on which diagnostic criteria are applied. For instance, when considering adult patients undergoing echocardiography, estimates range from 0.014% to 1.3%, while in heart failure populations, prevalence can reach 3–4% [12,14].

In children under age 10, the estimated incidence is 0.12 per 100,000, making LVNC the third most common pediatric cardiomyopathy after dilated and hypertrophic forms [13]. However, pediatric prevalence is difficult to define due to overlap with other cardiomyopathies and inconsistent application of diagnostic criteria. LVNC likely stems from a combination of genetic, developmental, and environmental factors, many of which are still being studied. Many cases of LVNC occur in families, and several genetic variants, particularly in sarcomeric and myocardial developmental genes like *MYH7* and *TTN*, have been linked to its expression. Familial LVNC cases are believed to constitute approximately 30–50% of instances, typically adhering to an autosomal dominant inheritance pattern, though X-linked recessive, autosomal recessive, and mitochondrial inheritance types have also been observed [15,16].

The clinical manifestations of LVNC reveal extensive variability, ranging from asymptomatic cases to severe presentations including heart failure, life-threatening arrhythmias, thromboembolic events, and sudden cardiac arrest, and even death [16,17]. The condition may manifest throughout an individual’s life, but it is primarily identified during infancy or early childhood. However, it remains uncertain whether this tendency for early onset indicates a more severe form of the disease or if it is merely a result of more vigilant monitoring. Gender distribution shows a male predominance, with men accounting for 52% to 86% of cases across various studies [18,19,20]. Some studies suggest ethnic variations in LVNC prevalence, with one study reporting a higher prevalence of myocardial hypertrabeculation in athletes of African descent (approximately 15% meeting echocardiographic criteria for LVNC) [18,21]. Again, it remains unclear whether this represents true LVNC or a physiological adaptation to increased cardiac preload [12,13,14,18,22,23].

## 5. Risk Factors

In certain patients, LVNC is part of a broader syndromic or structural cardiac context. LVNC has been reported alongside conditions such as Barth syndrome, Noonan syndrome, and Ebstein anomaly [12]. These presentations pose diagnostic challenges, especially when LVNC is one feature of a multisystem disorder. Interestingly, as many as 40% of patients diagnosed with LVNC have no identifiable genetic mutation or family history, raising the possibility that environmental influences or acquired mechanisms also play a role in its development and possess a viable risk factor [14,23]. This clinical spectrum, from familial and syndromic presentations to sporadic cases, makes it challenging to determine where true pathology ends and structural variation begins.

Adding further complexity, LVNC may not always be congenital. In adults without pathogenic mutations, hypertrabeculation has been observed in physiological states such as pregnancy, long standing hypertension, and athletic remodeling [23]. For example, pregnant women can develop de novo anatomical structural changes associated with LVNC which is attributed to increased preload. A longitudinal study followed pregnant women with normal echocardiographs throughout their pregnancy to show that only 25% of patients developed increased left ventricular trabeculations [11]. These cases suggest that some instances of LVNC reflect an adaptive morphological response to chronic preload elevation rather than a fixed developmental defect.

Pregnant women found to have hypertrabeculations were asymptomatic and had no reduction in ejection fraction and the majority of those, approximately 72%, had regression of trabeculations within the first 8 months postpartum, and most of the other women showed gradual cardiac remodeling and regression of myocardial abnormalities within 2 years [11]. Therefore, LVNC can be seen in pathological states where hypertrabeculation of the lower chambers are observed and coincide with other metabolic or genetic diseases. LVNC can also be physiologic and seen in certain instances where it reflects an intrinsically benign structural state with no link to other diseases.

Athletes, similar to pregnant women, can have physiological hypertrabeculations of the ventricles which also increases preload-associated conditions [11]. A research study looking at Olympic athletes showed that 1.4% had echocardiography findings that were consistent with LVNC, but only 0.1% (of the 1.4%) of those athletes had either an EF less than 50%, family history or genetic testing suggestive of LVNC [11]. The morphological structure of hypertrabeculations seen in athletes with LVNC is not known to have adverse effects [11].

The clinical significance of risk factors in LVNC patients remains relatively ambiguous, as many individuals are asymptomatic at the time of diagnosis, allowing potential for overdiagnosis and overtreatment [24]. This evolving understanding challenges traditional definitions and emphasizes the need for clinical context when interpreting imaging studies. Future classification systems and risk models must account for both inherited and acquired phenotypes, ideally informed by longitudinal studies capable of distinguishing pathological remodeling from physiological variation.

## 6. Genetics

LVNC can be genetically linked to both sporadic and familial forms and commonly inflicts the pediatric population with a minor subset of reports seen in adults, with high association between familial LVNC and neuromuscular disorders [25]. Approximately 12–50% of LVNC cases involve a family history with variable inheritance pattern including autosomal dominant, autosomal recessive, and X-linked inheritance [14]. The mosaic molecular pattern of LVNC’s origin resides in the pediatric population where 10–50% of patients have dysfunctions involving sarcomeric, cytoskeletal or ion channel protein dysfunctions, a majority caused by *MYH7*, *TTN*, or *MYBPC3* gene variants [26].

Utilizing next generation sequencing including gene panel or whole exome and classic Sanger sequencing, researchers in The Children’s Memorial Health Institute in Poland were able to discover 16 distinct genetic variants representing 11 out of 16 genes discovered from a total of 31 pediatric patients (52%), which includes 10 novel alterations [26]. They found that the most common genetic defects were found in genes: *HCN4* (*n* = 4), *MYH7* (*n* = 2), and *PRDM16* (*n* = 2) with discovery of other variants detected in genes: *ACTC1*, *ACTN2*, *HCCS*, *LAMA4*, *MYH6*, *RBM20*, *TAFFAZIN*, and *TTN* [26].

Other gene mutations that have been reported to cause LVNC solely in children include α-Dystrobrevin (*DTNA*), and G4.5 (*TAZ*) while genes: LIM domain-binding protein (*LDB3*, *Cypher/ZASP*), and Lamin A/C have been reported to cause LVNC in both children and adults [25]. Genes that cause LVNC in adults include sarcomere proteins such as: β-Myosin heavy chain (*MYH*7), α-Cardiac actin (*ACTC*), and Cardiac troponin T (*TNNT2*) [25]. Lack of cohesive communication between intricate protein units in the NOTCH signaling pathway has also been a well reported etiology and cause of LVNC in patients [27]. The genetics of LVNC cardiomyopathy are also intertwined with other cardiomyopathies. For example, Barth syndrome which affects the mitochondrial cardiolipin metabolic pathway has a variant form of ventricular noncompaction that is associated with a rapid and early-onset form of DCM and severe heart failure (HF) often leading to heart transplantation.

Since LVNC can be present in isolated form, concomitantly with other cardiomyopathies and metabolic disorders, in addition to appearing in physiologic states such as in pregnancy and in athletes where rates of complications are low, then it is evident that LVNC has been on the forefront of a common question: should individuals with LVNC be genetically tested for other cardiomyopathies or not? Research conducted within the pediatric population with LVNC has discovered that even in the cohort of patients with idiopathic LVNC at the time of presentation, 32% of those had familial disease and 9% had an underlying metabolic or syndromic genetic condition [28].

The answer to the million dollar question of whether genetic testing should be warranted in patients with LVNC sheds light on confirming the importance of discussing a broad list of differential diagnoses, especially in those patients that are diagnosed at a young age or those with a myriad or constellation of other clinical symptoms that are suspicious of an underlying or second “hit” cardiomyopathy, metabolic, sarcomeric, mitochondrial, or neuromuscular disease. LVNC may represent a benign anatomic physiologic variant in the complete absence of other cardiovascular disease findings [28]. The study data suggested a strong consideration of genetic testing for individuals with cardiomyopathy and co-occurring LVNC in addition to those individuals that have isolated LVNC and a family history of cardiomyopathy to further investigate is a second “hit” cardiomyopathy or a concomitant parallel cardiomyopathy along with isolated LVNC exists [28,29].

## 7. Pathophysiology

During the normal physiologic evolution of human development, particularly during the end of the 4th week of gestation, trabeculations appear in the cardiac jelly forming a spongy and porous structure which protrudes into the ventricular lumen. During this crucial stage in the gestational period, the thin subepicardial unit of the cardiomyocytes forms the “compacted myocardium” while the trabecular part forms the “non-compacted myocardium” that eventually thickens during the next several weeks thus, increasing the volume of the compacted layer while the intertrabecular spaces compress and fuse forming capillaries [30].

LVNC is caused by the disturbance of this process and the lack and/or dysfunction of this mechanism which leads to the existence of a hyper-trabeculated morphological state. LVNC can lead to the formation and development of LV dilation or hypertrophy, systolic and/or diastolic dysfunction, and if left untreated can cause an increased risk of left and/or right ventricular failure, ventricular arrhythmias, or complete atrioventricular block, sudden cardiac arrest, and even death [14,30]. Another argument to the pathophysiologic origin of LVNC in adults is primarily driven by the suspicion that this type of cardiomyopathy can be acquired later in life as a morphological trait associated with other types of cardiomyopathies [14].

## 8. Clinical Presentation & Complications

The clinical course of LVNC varies widely and is shaped by a complex interplay of structural, functional, and genetic factors. While many patients may remain asymptomatic for years, others may present with heart failure, arrhythmias, or thromboembolic events. Prognosis is diverse, where outcomes and phenotypes differ between adults and children, depending on development of specific complications.

### 8.1. Heart Failure

Heart failure is a common complication of LVNC, occurring in approximately 19–43% of patients, with increased prevalence among those with congenital heart abnormalities or genetic mutations [31,32,33]. The underlying mechanisms for heart failure stem from the excessive trabeculations and deep intertrabecular recesses, impairing myocardial contractility, relaxation, and ventricular compliance [34]. This abnormal myocardial architecture may induce inefficient myocardial fiber shortening, furthering progression of chamber dilation, ultimately presenting as clinical symptoms of dyspnea, peripheral edema, and exercise intolerance [34,35].

In adults, LVNC typically manifests as left ventricular systolic dysfunction, though advanced cases may involve both ventricles. Some patients remain compensated for years until decompensation is triggered by arrhythmias, volume overload, or secondary insults [36]. In pediatric populations, congestive heart failure is often the initial presentation, frequently occurring alongside mixed cardiomyopathic phenotypes such as dilated or hypertrophic forms [37]. These clinical features may also correlate with underlying pathogenic variants, particularly in genes encoding sarcomeric or cytoskeletal proteins [12].

### 8.2. Arrhythmias

Arrhythmias are a key clinical concern in patients with LVNC and are often implicated in increased morbidity and mortality. Patients with LVNC may exhibit various forms of arrhythmias, including ventricular tachycardia (VT), ventricular fibrillation (VF), atrial fibrillation (AF), supraventricular tachycardia (SVT), left bundle branch block (LBBB), atrial tachycardia (AT), atrial premature contractions (APC), sick sinus syndrome (SSS), right bundle branch block (RBBB), ventricular premature contraction (VPC), atrioventricular block (AVB), and malignant arrhythmias (MA) [38].

Among the most critical of the rhythm disorders related to LVNC are the ventricular tachyarrhythmias, including monomorphic VT, bidirectional VT, and polymorphic VT and VF [12,24]. VT and VF are a leading cause of sudden cardiac arrest in LVNC in both children and adults, and their presence alone is an independent risk factor for mortality [15,39,40]. The risk associated with malignant arrhythmias can be unpredictable, as one study demonstrated 36% of patients with early-stage LVNC developed malignant ventricular arrhythmias in a 5-year follow-up period [41]. AVB is an important but less common conduction abnormality in patients with LVNC as it has been shown to induce hemodynamic compromise, presenting clinically as syncope or sudden death.

Supraventricular arrhythmias such as atrial fibrillation and atrial tachycardia may also occur in LVNC and are more prevalent in patients with left atrial dilation or impaired diastolic filling. These rhythm disturbances not only complicate the hemodynamic profile of affected individuals but also increase the likelihood of thromboembolic events, particularly in the setting of coexistent systolic dysfunction or left atrial stasis [42,43]. Electrocardiographic abnormalities are frequently observed in patients with LVNC, including repolarization changes, intraventricular conduction delays, and frequent premature ventricular contractions. However, there is no pathognomonic ECG (electrocardiogram) pattern, and rhythm monitoring remains essential for risk stratification.

### 8.3. Thromboembolic Events

The risk of thromboembolic events is a pressing concern in patients with LVNC, as such, it is essential to consider the implications of stasis in the deep intertrabecular recesses, particularly in those with reduced left ventricular function. The stagnant blood flow in these areas can lead to clot formation, which poses a significant risk for complications such as stroke and systemic embolization, especially in the context of concurrent arrhythmias or structural abnormalities [42,44]. The presence of left ventricular thrombus (LVT) further complicates the clinical management of these patients. LVT is observed in approximately one in six cases of left ventricular systolic dysfunction and is particularly concerning due to its association with increased stroke risk and systemic embolism [45,46].

## 9. Prognosis

LVNC has a highly variable prognosis, with outcomes dependent on heart function, degree of trabeculations and noncompaction, and presence of symptoms at time of diagnosis. Population-level studies demonstrate reduced five-year survival compared to age- and sex-matched controls, supporting the increased morbidity and mortality associated with LVNC [47]. Additional studies have identified reduced left ventricular ejection fraction (LVEF < 50%), advancing age, and the presence of ventricular tachycardia as independent predictors of major adverse cardiac events (MACE) [48].

A recent meta-analysis found that NYHA (New York Heart Association) heart failure symptoms, elevated NT-proBNP, decreasing LVEF and increasing LV end-diastolic diameter were significantly associated with increased risk of MACE [49]. In order to better understand and operationalize these risk factors, clinical scoring systems have been developed to aid in risk stratification and disease monitoring over time. For instance, the recently proposed ABLE score integrates age, NT-proBNP, LVEF, and LV end-diastolic diameter into a simplified predictive model for adverse cardiovascular outcomes, showing strong discriminatory power in both derivation and validation cohorts [50]. Such tools may be valuable in guiding surveillance strategies and therapeutic decisions in patients with LVNC.

While LVNC is a clinically and genetically heterogeneous condition, genotype-phenotype correlation is emerging as valuable in prognostications and care. Distinguishing genetics from non-genetic non-compaction cardiomyopathy (NCCM) might complement risk prediction and subsequently guide management of patients with follow-up tailored to genetic status. For example, studies have demonstrated adverse cardiac events occur more often in mutation carriers with left ventricular systolic dysfunction than in sporadic cases [37]. Although some studies suggest that the degree of noncompaction may serve as an independent prognostic factor, incidental findings of excessive trabeculation in adults with preserved systolic function and normal myocardial morphology are typically managed according to the presence or absence of clinical symptoms rather than imaging alone [36].

Beyond clinical and structural features, cardiac structural parameters also offer prognostic value. The prognostic significance of left ventricular diastolic volume has been extensively studied, with the Framingham Study establishing its correlation with adverse clinical outcomes. The prognostic value of trabeculation appears to be controversial, as initial studies of patients with dilated cardiomyopathy found that the degree of trabeculation was not independently predictive of MACE [51]. Additionally, global longitudinal strain has been shown as a stronger predictor of cardiovascular outcomes compared to trabecular mass, when studied in patients with a noncompaction phenotype [52]. However, the inherent anatomical relationship between diastolic volume, trabecular mass, and free volume suggests that each component may contribute differently to risk stratification.

Separate analyses have indicated that left ventricular trabecular mass provides incremental prognostic value beyond diastolic volume alone, and carries more prognostic information than free volume [53]. These findings imply that the clinical relevance of left ventricular diastolic volume may be influenced by the extent of trabecular development, potentially offering a more comprehensive indicator of cardiac function and risk for complications such as malignant arrhythmias and thromboembolic events. Tissue characterization using cardiac magnetic resonance imaging offers additional prognostic value. Late gadolinium enhancement (LGE) is a marker of myocardial fibrosis and has been associated with poor outcomes in LVNC; the presence of LGE has been independently linked to increased risk of MACE, including HF hospitalization, sustained ventricular arrhythmias, and sudden cardiac death, even after adjusting for LVEF and chamber size [54]. Together, these findings highlight the importance of integrating functional, structural, and tissue-level data for more accurate risk stratification in LVNC.

## 10. Imaging Studies

**Figure 2:**
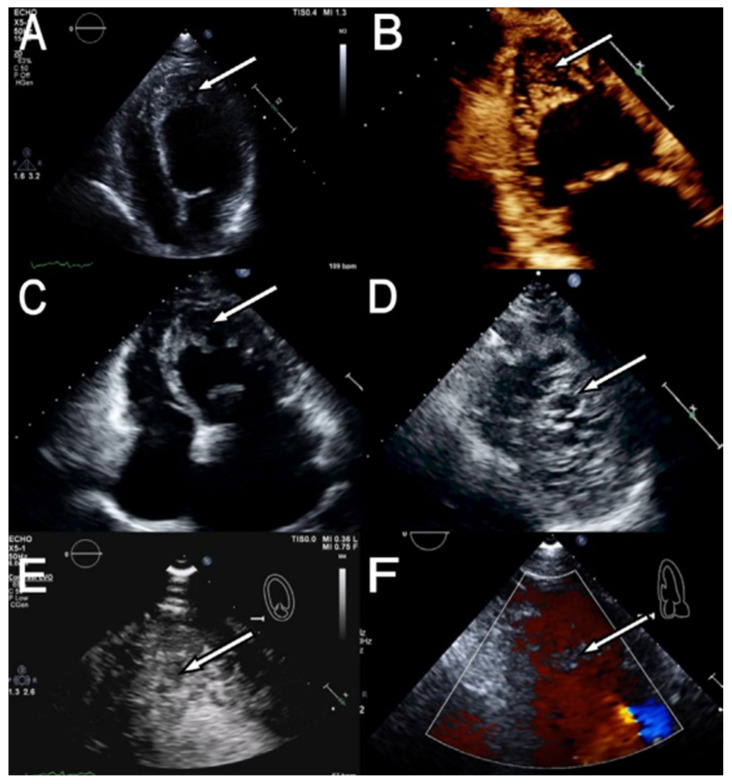
Transthoracic echocardiographic imaging views of a patient with LVNC. (**A**)—Apical 4-chamber view showing apical noncompaction (*arrow*). (**B**)—Apical 2-chamber view showing apical noncompaction (*arrow*). (**C**)—Apical 4-chamber view showing LVNC (*arrow*). (**D**)—Parasternal short-axis view at the level of the LV apex showing noncompaction (*arrow*). (**E**)—High magnification of LV apex showing noncompaction highlighted by contrast (*arrow*). (**F**)—Color Doppler flow within intertrabecular recesses shown in LVNC (*arrow*). *Source: Rao, K., Bhaskaran, A., Choudhary, P., & Tan, T. C. (2020). The role of multimodality imaging in the diagnosis of left ventricular noncompaction. European Journal of Clinical Investigation, 50(9), e13254* [55].

Imaging modalities utilized to diagnose LVNC include traditional echocardiography (Figure 2), cardiac computed tomography (CCT), and cardiac magnetic resonance (CMR) imaging with the latter becoming more accessible as a diagnostic technique for LVNC due to its lack of radiation when compared to CCT with contrast [6]. However, both CMR and CCT are limited in the neonate and pediatric population because this population is unable at times to follow directions especially when the patient needs to be absolutely still to avoid imaging fragmentation and spatial convolution during these studies to provide the clinician with the best possible visual results needed for an accurate diagnosis. In adults, echocardiographic criteria, speckled tracking, and CMR has been utilized for the confirmation diagnosis of LVNC [55]. The biggest issue with diagnostic imaging studies in LVNC is the vast array of variability that exists amongst these different modalities.

While CMR is considered the gold standard for the initial evaluation of the hypertrabeculation morphology seen in the LV, the optimal imaging modality for follow-up evaluation remains uncertain [56]. For example, in a study investigating correlation agreements among two-dimensional transthoracic echocardiography (2D_TTE), three-dimensional transthoracic echocardiography (3D_TTE), and CMR amongst 38 LVNC subjects in order to calculate and interpret volumetric and strain parameters became difficult. These 38 LVNC subjects with indexed end-diastolic, end-systolic, and stroke volume, ejection fractions, and global longitudinal and circumferential strains showed lower correlation and higher percentage of errors compared to their healthy (non-LVNC) counterparts [56]. The research concluded that while echocardiography is suitable for volumetric follow-up in LVNC after baseline CMR evaluation, the deformation parameters are not interchangeable between modalities due in part to the trabecular interference and disruption that the non-compacted myocardium is responsible in causing through the visual spectrum [56].

Overdiagnosis of LVNC causes an unwarranted exaggeration in an attempt to label a particular cardiomyopathy something that it truly is not. Unfortunately, there is no dedicated genetic or laboratory test for the diagnosis of LVNC that is highly sensitive and highly specific [57]. Furthermore, LVNC can mimic other cardiomyopathies for example, in myocarditis the remodeling of the heart can mimic morphological changes that appear to look and mimic LVNC on echocardiography studies [57]. It is important to obtain a thorough and accurate patient history as prior cardiac diseases can possibly invalidate the diagnosis of LVNC as other concomitant cardiac abnormalities will essentially disqualify the diagnosis of LVNC [57].

### 10.1. Echocardiography

Echocardiography is still the most common imaging modality utilized to diagnose and describe the findings of LVNC in the neonate population. Transthoracic echocardiography (TTE) is low risk, cost-effective and easily accessible, especially to nations where healthcare funding is low and the demographic population impacted by this cardiomyopathy remains impoverished. The landmark paper from pioneers Jenni et al. highlighted the utilization of echocardiographic and pathoanatomical characteristics of isolated ventricular non-compaction (IVNC) cardiomyopathy for the sole goal of classifying this cardiomyopathy. The researchers utilized measurements that they calculated in the parasternal short-axis view in the end-systole phase of the cardiac cycle. Four crucial morphological criteria to diagnose IVNC were discovered [58]:

1. The absence of coexisting cardiac abnormalities.
2. A double layer structure was visualized: a thin compacted epicardial band and a thick non-compacted endocardial layer consisting of trabecular meshwork with deep endomyocardial spaces.
3. Predominant location of the pathological defect was primarily the mid-lateral region, then apical, and lastly mid-inferior.
4. Color Doppler evidence solidified the visualization of deep perfused intertrabecular recesses.

Gebhard and other fellow researchers added to the work of Jenni et al. findings with a retrospective study comparing the myocardium of LVNC patients with at least moderate aortic stenosis, which per the American College of Cardiology is defined as peak aortic jet velocity between 3 to 3.9 m/s, mean pressure gradient between 20–39 mm Hg, and aortic valve area between 1.0–1.4 cm^2^ and discovered that compacted myocardium with an absolute value less than 8 mm can further help in differentiating LVNC cardiomyopathy [55,59]. Another retrospective study analyzing digital planimetry for accurate quantification in LVNC patients proposed severity of non-compaction under an apical four-chamber TTE (systole or diastole) with a total non-compaction area of up to 2.5 cm^2^ equating to a mild case, 2.5–5 cm^2^ being moderate and greater than 5 cm^2^ suggestive of severe LV non-compaction [60].

Other major criteria that complemented with the works of Jenni et al. included criteria proposed by Stollberger and colleagues which consisted of quantifying the number of trabeculations in close proximity to the papillary muscles being highly specific for LVNC while utilizing Doppler imaging or presence of echo enhancing agent within the intertrabecular recesses to distinguish these findings [61]. Another study review investigating LVNC in patients found the importance of anatomical distribution of hypertrabeculations as being just as specific for diagnosing LVNC by mapping the presence of these trabeculations in relation to cardiac zones (i.e., apical, inferior and lateral) [62]. Advanced TTE techniques can greatly improve sensitivity and specificity of diagnosing LVNC, especially when utilizing 3D TTE, or by usage of contrast such as Definity^®^ or Optison which shows higher spatial resolution for determination of non-compacted myocardium in regions including the apex and mid-ventricular LV regions [55].

Recent findings include a maximal end-systolic ratio of NC:C > 2 and deep intertrabecular recesses with a predominant location in the cardiac apex [1,7]. While utilizing echocardiography, Børresen et al., showed that LV ejection fraction was decreased in the neonate population when compared to the matched control groups [9]. In asymptomatic neonates, echocardiography still has shown impaired LV systolic function in the setting of hyper-trabeculations in LVNC [7]. The use of Doppler color, myocardial colorization, and assessment of all apical segments, can also improve the visualization and subsequent identification of the hyper-trabeculations seen in the non-compact layer of the LV as well as the deep intertrabecular recesses that are appreciated using this imagery [7].

Some limitations of TTE in diagnosing LVNC include overall poor visualization of the LV apex when compared to other imaging modalities in addition to operator dependence and patient variables including body habitus and chest wall deformity complicated with perhaps other concomitant genetic neuromuscular diseases that patients might have [55]. Other limitations can include overestimation of trabecular morphology in the LV if the TTE is not exactly perpendicular to the long axis of the LV or if there is slight characteristic changes in hypertrabeculations that can mask and impact the image resolution of other adjacent structures [55].

The current criteria in diagnosing LVNC also falls short if the same imaging modality is attempted to be utilized to assess right ventricular non-compaction (RVNC) as these guidelines cannot be validated when discussing the RV as it exhibits more prominent trabeculations [55]. TTE should be utilized in conjunction with cardiac magnetic resonance (CMR) imaging, transesophageal echocardiography (TEE) or cardiac CT (CCT) in order to present a more definitive LVNC diagnosis. For example, a comprehensive review discovered that CMR with late gadolinium enhancement was superior for detection of LV thrombi that occurs in higher percentages as a consequence to deep trabeculations in the LV with a sensitivity of 88% and specificity of 99% compared to TTE with contrast [63].

### 10.2. Cardiac Magnetic Resonance

One inherent limitation to echocardiography in the diagnostic process of LVNC is the low visual quality that it provides in respect to the ventricular apex when compared to CMR, which shows unremarkable details, resolution, and overall appearance of the ventricular apex in great clarity over traditional echocardiography. Petersen et al., discovered that the degree of LVNC cardiomyopathy was more frequent amongst previously labeled healthy, dilated, and hypertrophied hearts when these cases were analyzed under CMR due to the high sensitivity [64].

**Figure 3:**
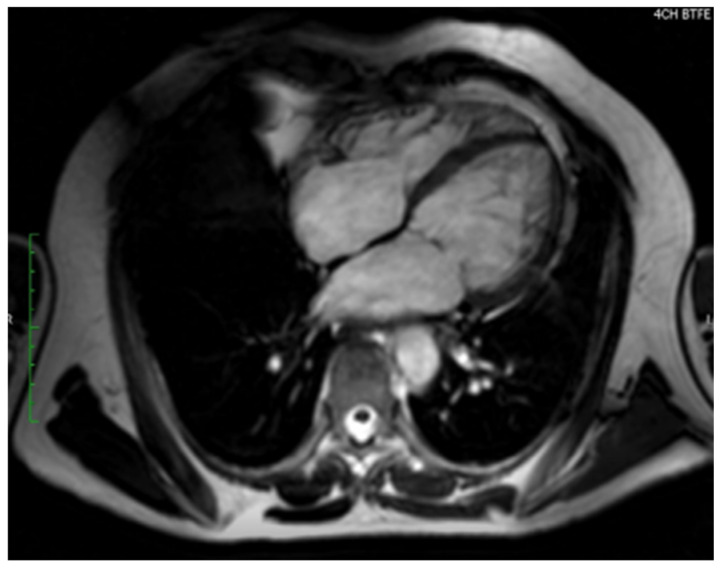
Axial view of cardiac magnetic resonance (CMR) imaging depicting biventricular myocardial noncompaction. *Source: Rao, K., Bhaskaran, A., Choudhary, P., & Tan, T. C. (2020). The role of multimodality imaging in the diagnosis of left ventricular noncompaction. European Journal of Clinical Investigation, 50(9), e13254* [55].

The advantage of CMR when measuring cardiac-hemodynamic metrics in LVNC patients (Figure 3) is from the result of superior imaging quality along with the accurate calculation of thickness ratios of the trabecular and compact layers during the diastolic steady-state of the heart [64]. The diastolic ratio given with high diagnostic accuracy for LVNC observed in previous healthy, dilated, and hypertrophied hearts has a slightly higher cut-off value of >2.3 when compared to echocardiography (i.e., NC:C > 2) which is taken during systole [64].

Similar to the TTE criteria by Jenni et al. for LVNC, there have been various comprehensive reviews on CMR criteria and LVNC diagnosis. One of the early pioneers that studied CMR utility amongst LVNC patients, specifically imaging in the short-axis plane of end-diastole, discovered the value and criteria of that trabeculations inside the LV mass > 20% of total mass is diagnostic for LVNC with a sensitivity of 91.6% and specificity of 86.5% [65]. Short-axis end-diastolic phase CMR imagery was also used to visualize LV trabeculations with a volume of >35% of the total LV volume with a sensitivity of 66.1% and a specificity of 89.7% [66].

Another paper emphasizing CMR criteria specific for LVNC diagnosis also discovered morphological changes by looking at the heart in the short-axis imaging plane and during the end-diastole phase of the cardiac cycle. This investigation highlighted the findings that trabeculations inside the LV being >25% of the total LV mass, while trabeculations in the LV mass in ratio to body surface area (BSA) > 15 g/m^2^ were diagnostic of LVNC [67]. This study also mentioned that NC:C > 3 in certain imaging segments was diagnostic of LVNC with a sensitivity of 75% and specificity of 100% [67].

Between 2008 and 2022 in a large tertiary center in Warsaw, Poland a study was conducted to determine the impact of CMR on the diagnosis of LVNC after known or suspicions of LVNC were first imaged by TTE. Amongst the study sample of 333 patients, 193 (58.0%) being male with a median age of 39.0 (26.8–51.0) years, researchers from this institution discovered that out of the 74 patients that fulfilled the TTE LVNC criteria, the diagnosis was confirmed in 54 (73.0%) of cases while utilizing CMR [68]. The study concluded that CMR utilization in the diagnostic accuracy and differentiation of LVNC and other cardiac diseases was extremely valuable.

A cross-sectional cardiovascular screening study for sudden cardiac arrest from 5169 middle and high school students with a mean age of 13.1 +/− 1.78 years showed that pathologically excessive, and even asymptomatic, LV trabeculations can be associated with unsustainable physiologic disadvantage that would essentially increase the risk of LV dysfunction, pathologic remodeling, arrhythmias, or mural thrombi [69].

The limitations to CMR in diagnosis of LVNC include the excessive costs and it being less readily available especially in nations with low healthcare budgets and high disease burden due to poor monitoring, surveillance, and lack of medical prevention guidelines. Black blood imaging used as a technique to visualize the walls of blood vessels in the hopes of demonstrating better visualization for pathologies such as blood vessel wall thickening, inflammation, or plaque burden has presented a challenge in CMR LVNC diagnosis. This challenge is driven from the stagnant blood that is caused by decreased flow from interactions with myocardial trabeculations leading to pseudo thickening of the ventricular wall, causing either a LVNC to be misdiagnosed or overlooked [25].

### 10.3. Cardiac Computed Tomography

**Figure 4:**
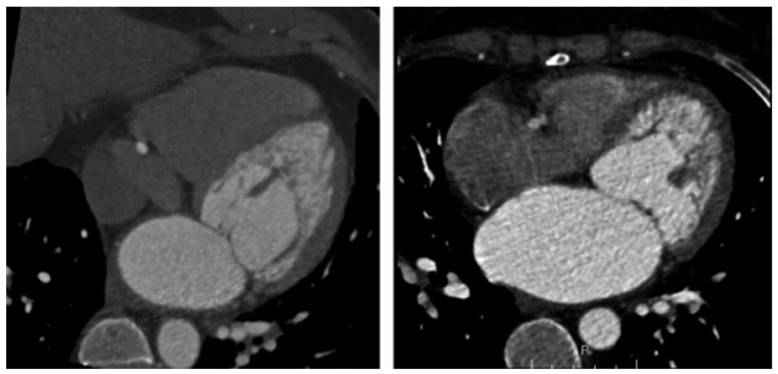
Axial view of cardiac computed tomography (CCT) imaging with coronary angiography showing LVNC. *Source: Rao, K., Bhaskaran, A., Choudhary, P., & Tan, T. C. (2020). The role of multimodality imaging in the diagnosis of left ventricular noncompaction. European Journal of Clinical Investigation, 50(9), e13254* [55].

Cardiac computed tomography (CCT) has become a new and innovative imaging modality in diagnosing LVNC and similar to its competitors, like TTE and CMR, it provides viewers with high spatial quality visualization to delineate the characteristic two-layered myocardium with prominent trabeculae (Figure 4). Two landmark papers utilizing CCT imaging proposed the diagnostic criteria for LVNC cardiomyopathy albeit both studies had relatively small sample sizes but compared LVNC patients to healthy controls as well as other etiologies of cardiomyopathy [55].

Both of these studies were retrospective and examined LVNC patients who had undergone standard CT coronary angiography with electrocardiographic gating to rule out coronary artery disease. They measured NC/C ratios in the short-axis plane at multiple ventricular levels and concluded that LVNC diagnosis can be just as reliable as NC/C cut-offs used in CMR with values of 2.2 and 2.3 taken at the end-diastole phase greater than 2 myocardial segments [70,71].

An in-depth systematic review and meta-analysis discovered the valuable and highly sought after imaging tool and impact that CCT has on LVNC diagnosis. This overview discovered the relationship between NC/C ratio of ≥1.8 as a diagnostic criterion which represents a giant step toward potentially improving LVNC identification and detection while mitigating the challenges of underdiagnosis or misdiagnosis amongst the population afflicted [72]. The more advanced form of CCT termed multidetector computed tomography (MDCT) can also be utilized to diagnose LVNC.

MDCT scans can identify and show the various layers of LV myocardial tissue, specifically the non-compacted and the compacted layer, along with providing the metrics to assess for LV functionality [73]. The limitations of MDCT in LVNC diagnosis and management, when compared to CMR, is that MDCT has a difficult time accurately depicting areas of fibrosis within the cardiac tissue leaving patients vulnerable to acquiring deadly arrhythmias [73]. MDCT is superior to both CMR and TTE in examining the coronary arteries which can exclude coronary artery disease in LVNC patients.

A study utilized ultrafast CT, also known as electron-beam CT (EBCT), which is able to detect and capture detailed cardiac images during a single heartbeat when compared to traditional CCT. In the study, Ultrafast CT, CMR, and TTE were tested to evaluate for the anatomical and pathological diagnosis of isolated noncompaction of the LV in six patients (three sets of siblings) ranging in age from 13–18 years. The study concluded that ultrafast CT or EBCT and CMR provided the viewer with high-resolution imaging of the non-compacted LV myocardium while also offering specific pathophysiologic details regarding this disease when both of these imaging tools were compared to traditional TTE [74].

While CCT and its variants such as EBCT and MDCT provide exceptional imaging power in LVNC diagnosis, the achilles heel of these tools include the unwanted risk and exposure to radiation which should be limited in the pediatric population and as such traditional TTE and CMR are favored more than CCT. With the addition or supplementation of contrast in these particular CCT imaging variants, this causes another layer of iatrogenic complication, the risk of acute kidney injury and a worsening renal function. Thus, even though CCT, EBCT, and MDCT have their tremendous advantages in LVNC diagnosis, nothing is without risk and likewise can be said about TTE and CMR, as CCT has its own inherent flaws. A summary of pertinent diagnostic imaging studies (Table 2) utilized to distinguish LVNC is shown.

## 11. Treatment

Management of left ventricular non-compaction cardiomyopathy (LVNC) remains largely supportive and symptom-driven, given the heterogeneity in clinical presentation and the absence of disease-specific, evidence-based guidelines. As such, treatment strategies typically align with established approaches for heart failure, arrhythmia surveillance, and thromboembolism prevention similar to other cardiomyopathies [12,72,73,75].

### 11.1. Heart Failure Management

In patients with reduced ejection fraction, guideline-directed medical therapy (GDMT) for heart failure with reduced ejection fraction (HFrEF), including beta-blockers, renin-angiotensin system inhibitors and receptor blockers (ACEi or ARBs) or angiotensin receptor-neprilysin inhibitors (ARNIs), mineralocorticoid receptor antagonists (MRAs), and sodium-glucose co-transporter 2 (SGLT2) inhibitors, is commonly applied, with treatment regimen tailored to tolerance and clinical response [12,52]. Mechanical circulatory support or cardiac transplantation may be considered in patients with refractory heart failure. In pediatric populations, particularly those with biventricular involvement or overlapping dilated phenotypes, medical therapy remains the cornerstone, though transplantation is pursued more frequently due to a more aggressive clinical course [37].

### 11.2. Arrhythmia Surveillance & Device Therapy

Given the increased risk of malignant arrhythmias and sudden cardiac arrest, continuous rhythm monitoring is recommended in symptomatic individuals or those with high-risk features, such as severely reduced LVEF, documented non-sustained ventricular tachycardia, or family history of sudden death. Implantable cardioverter-defibrillator (ICD) therapy may be indicated in selected patients based on conventional criteria for primary prevention in HFrEF or for secondary prevention in those with prior ventricular arrhythmias [76]. The presence of late gadolinium enhancement on cardiac MRI has also been associated with increased arrhythmic risk, though its role in decision-making for ICD implantation remains under investigation [61]. CMR with LGE is sensitive and specific for identifying cardiac fibrosis which can foster an environment with poor conduction system abnormalities requiring ICD implantation [77].

### 11.3. Anticoagulation & Thromboembolism Prevention

The highly trabeculated myocardium of LVNC has been proposed as a potential substrate for thrombus formation. However, prophylactic anticoagulation in all patients with LVNC is not universally recommended. Instead, anticoagulation is typically reserved for patients with atrial fibrillation, reduced LVEF, intracardiac thrombus, or prior thromboembolic events [12,78]. The optimal antithrombotic strategy remains uncertain, with some studies suggesting elevated thromboembolic risk even in the absence of traditional risk factors. Patients with increased risk of thromboembolic events such as those with prior CVA would benefit from therapeutic anticoagulation. Current studies justify the usage of prophylactic therapeutic anticoagulation in LVNC with low EF (EF < 40%) and/or atrial fibrillation [79]. Similar research also provides evidence of anticoagulation use in presence of thrombus caused by hypertrabeculations of the left ventricle either in physiologic LVNC [79].

### 11.4. Genetic Counseling & Family Screening

Given the growing body of evidence linking LVNC to pathogenic variants in sarcomeric, cytoskeletal, and mitochondrial genes, genetic testing is recommended in patients with familial cardiomyopathy or early-onset disease. Identifying a causative mutation can inform prognosis, guide family screening, and clarify the distinction between isolated LVNC and phenotypic overlap with other cardiomyopathies [37].

### 11.5. Multidisciplinary Approach

Due to the phenotypic variability of LVNC, a tailored, multidisciplinary approach is essential. Cardiac imaging specialists, electrophysiologists, heart failure clinicians, and genetic counselors all contribute to comprehensive care. Continued investigation is needed to define which features of LVNC predict progression to symptomatic disease and to develop risk-adapted algorithms for surveillance and intervention.

## 12. Conclusions

Respectfully, even in the setting of advisors and under the guidance of academic institutional giants such as the American Heart Association (AHA), the European Society of Cardiology (ESC), and the European Working Group of Myocardial Disease feuding over the nomenclature and categorization of LV non-compaction cardiomyopathy, one thing is agreed upon, LVNC is a devastating illness with severe repercussions and negative implications on cardiovascular health. Without adequate imaging studies such as TTE, CMR, and CCT to diagnose LVNC, one cannot discover all of the possible medical and pharmacological therapies that are available. Epidemiology, environmental factors, and genetics all play a crucial role in LVNC and its subsequent management. Even though as many as 40% of patients diagnosed with LVNC have no identifiable genetic mutation or family history, raising the possibility that environmental influences or acquired mechanisms can be just as important makes the subject of LVNC cardiomyopathy of great importance. LVNC brings upon severe complications that include heart failure, deadly arrhythmias like VT or VF, and thromboembolic events which can progress to cerebrovascular accidents (CVA) if not treated.

Whether LVNC is a “true” cardiomyopathy or only a mere structural variant is only a point of discussion. Further research and clinical investigations should be conducted to solidify its importance similar to new concepts surrounding atrial cardiomyopathy (AtCM), the progressive structural disease of the atrial myocardium that can cause arrhythmias such as AF [80]. Thus, newer guidelines and an evidence-based framework should be developed to provide more insight into the world of cardiomyopathy for the sole purpose of shedding light on this interesting cardiac finding. This review plays a pivotal role in answering the vast amounts of questions that have previously went unanswered in this field by diving into LVNC cardiomyopathy as not only a mere argumentative instrument tool for research titans to disagree upon, but as a functional, living, and breathing entity that has tentacles much like the trabeculations seen inside the left ventricle.

## Figures and Tables

**Table 1:** Nine phenotypic heterogeneities of LVNC subtypes.

**Type I**	LVNC with abnormal LV hyper-trabeculation in isolation
**Type II**	LVNC with arrhythmia (arrhythmogenic cardiomyopathy)
**Type III**	LVNC with dilated cardiomyopathy (DCM)
**Type IV**	LVNC with hypertrophic cardiomyopathy (HCM)
**Type V**	LVNC associated with features consistent with HCM + DCM, or HCM or DCM with features of restrictive physiology
**Type VI**	LVNC with dilated atria and diastolic dysfunction
**Type VII**	LVNC with biventricular cardiomyopathy in both the LV & RV
**Type VIII**	Right Ventricular Non-Compaction (RVNC) with normal LV
**Type IX**	CHD form of LVNC

**LVNC** = left ventricle non-compaction, **DCM** = dilated cardiomyopathy, **HCM** = hypertrophic cardiomyopathy, **RV** = right ventricle, **RVNC** = right ventricle non-compaction, **CHD** = congenital heart disease.

**Table 2:** Summary of various diagnostic imaging modalities utilized to distinguish LVNC.

Author	Method	Diagnostic Criteria	Cardiac Phase	Cut-Off Limits	Strengths	Limitations
Jenni	ECHO	Ratio of compacted & non-compacted endocardium. Absence of coexisting cardiac abnormalities, the presence of deep trabeculations, which are filled with blood	Short axis, End systole	NC/C ≥ 2	Universal clinical acceptance, End-systolic measurement, Imaging validation using NC:C > 2.0	Elevated risk of overdiagnosis, Operator variability equating to low reproducibility, Difficult to distinguish subtypes
Petersen	CMR	Ratio of compated epicardium and non-compacted endocardium	End diastole	NC/C ≥ 2.3	Optimum imaging quality, High sensitivity, Widespread clinical usage	Poor prognostic correlation, Lower specificity based on quantifiable metrics, High rate of overdiagnosis
Jacquier	CMR	A value of trabeculated LV mass above 20% of the global mass of the LV	End diastole	LV trabeculated mass > 20%	Advantages inclduing diagnostic accuracy, Robust and reproducibility when compared to Petersen’s method, Interobserver reproducibility, Quantitative and objective measurements based on global LV mass	Time-consuming, Potential for overdiagnosis in healthy patients (i.e. pregnancy, athletes), Risk of overestimation mistake of calculating papillary muscle inclusion
Grothoff	CMR	Ratio of total LV trabeculated mass to global myocardial mass	End systole	Trabeculated ventricular mass greater than 25% of the global left ventricular mass; noncompated mass greater than 15 g/m^2^	High reduction in overdiagnosis with specificity near 100% for detection of true LVNC compared to mistakenly classifying healthy individuals or those with other cardiomyopathies (e.g., hypertrophy) as having LVNC	Too complex and time-consuming. The high specificity might make the criteria stringent and lead to low sensitivity. CMR dependence limits its uses as financial costly.
Melendez-Ramirez	MDCT	Ratio of compated and non-compacted endocardium in at least 2 or more segments	All 17 segments, end diastole	NC/C ratio > 2.2	High diagnostic accuracy with high sensitivity and specificity in accurately distinguishing LVNC from other cardiomyopathies. MDCT provides superior visualization of apical region when compared to traditional TTE allowing for possible 3-dimensional capabilities.	Radiation exposure based on imaging choice, Lower rate of ability to detect fibrosis unlike CMR with late gadolinium enhancement (LGE). The original study had a small sample size which can limit its generalized population applicability.

*Adapted: Paluszkiewicz, J., Milting, H., Kałużna-Oleksy, M., Pyda, M., Janus, M., Körperich, H., & Piran, M. (2022). Left ventricular non-compaction cardiomyopathy-still more questions than answers. Journal of Clinical Medicine, 11(14), 4135* [30].

## Data Availability

No new data were created or analyzed in this study.

## Abbreviations

**LVNC**	Left-ventricular non-compaction
**CHD**	Congenital heart disease
**TTE**	transthoracic echocardiography
**LV**	Left ventricle
**NC**	Noncompacted
**C**	Compacted
**AHA**	American Heart Association
**RV**	Right ventricle
**ESC**	European Society of Cardiology
**SCA**	Sudden cardiac arrest
**SCD**	Sudden cardiac death
**DCM**	Dilated cardiomyopathy
**HCM**	Hypertrophic cardiomyopathy
**ICD**	Implanted cardiac defibrillator
**EF**	Ejection fraction
**RVNC**	Right-ventricular non-compaction
**HF**	Heart failure
**VT**	Ventricular tachycardia
**VF**	Ventricular fibrillation
**AF**	Atrial fibrillation
**SVT**	Supraventricular tachycardia
**LBBB**	Left bundle branch block
**AT**	Atrial tachycardia
**APC**	Atrial premature contraction
**SSS**	Sick sinus syndrome
**RBBB**	Right bundle branch block
**VPC**	Ventricular premature contraction
**AVB**	Atrioventricular block
**MA**	Malignant arrhythmias
**ECG**	electrocardiogram
**LVT**	Left ventricular thrombus
**LVEF**	Left ventricular ejection fraction
**MACE**	Major adverse cardiac events
**NYHA**	New York Heart Association
**NCCM**	Non-compaction cardiomyopathy
**LGE**	Late gadolinium enhancement
**CCT**	Cardiac computed tomography
**CMR**	Cardiac magnetic resonance
**IVNC**	Isolated ventricular non-compaction
**NC:C**	Non-compaction to compaction ratio
**TEE**	Transesophageal echocardiography
**BSA**	Body surface area
**MDCT**	Multidetector computed tomography
**EBCT**	Electron-beam computed tomography
**GDMT**	Guideline-directed medical therapy
**HFrEF**	Heart failure with reduced ejection fraction
**ACEi**	Angiotensin converting enzyme inhibitor
**ARB**	Angiotenin receptor blocker
**ARNI**	Angiotenin receptor-neprilysin inhibitor
**MRA**	Mineralocorticoid receptor antagonist
**SGLT2**	Sodium-glucose co-transporter 2
**CVA**	Cerebrovascular accident
**AtCM**	Atrial cardiomyopathy
